# Identifying rare variants associated with hypertension using the C-alpha test

**DOI:** 10.1186/1753-6561-8-S1-S56

**Published:** 2014-06-17

**Authors:** Anna Faino, Amber Powell, André Williams, Lori Silveira

**Affiliations:** 1 Department of Biostatistics and Bioinformatics, National Jewish Health, 1400 Jackson Street, Denver, Colorado 80206, USA

## Abstract

Important rare variants may be near significantly associated common variants based on genetic distance. For this reason, we conducted an analysis of rare variants informed by tests of single-marker association at loci with common variants. We identified highly significant common variants within chromosome 3, as well as rare variants around these locations. Based on a predetermined window size, we then analyzed these rare variants with the C-alpha test to determine significant associations with hypertension. We found significant rare variants around common variants; however, the C-alpha test was sensitive to the specified window size. When comparing markers in genes to markers not in genes, we found that markers not in genes had more significant C-alpha test *p *values than markers in genes.

## Background

Whole genome sequencing provides geneticists and statisticians with the genome data necessary to attribute genetic variants with specific phenotypes such as high cholesterol, cancer, and diabetes. Many genome-wide association studies (GWAS) link phenotypes to genetic variants through logistic regression analyses. These single-marker association tests perform well for common variants (CVs). However, for rare variants (RVs), defined here as having minor allele frequencies (MAFs) of less than 0.05, single-marker association tests lack the power to detect significant associations [1].

Complex phenotypes have been found to be poorly explained by CVs. A hypothesis has emerged that RVs may contribute more significantly to disease heritability than CVs [2]. However, how to study these RVs has not been clear. Researchers have created various methods to statistically analyze RVs based on the idea of pooling together many RVs to increase statistical power. For many of these methods, subjects are either coded as having at least 1 RV or no RVs, or are coded based on a count of the number of RVs they have. Statistical analyses such as the cumulative minor-allele test (CMAT) and kernel-based adaptive clustering (KBAC) are powerful at detecting significance; however, their power diminishes in the presence of protective and harmful RVs [3-5]. The C-alpha test provides a computationally simple method for testing the significance of a set of RVs that can be protective, harmful, or neutral [6]. In particular, the C-alpha test assesses the following hypotheses:

H_o_: *p_i _*= *p_0_*

H_a_: *p_i _*follows a mixture distribution, with some variants detrimental (*p_i _*>*p_0_*), some neutral, and some protective (*p_i _*<*p_0_*)

where *p_i _*is the proportion of the rare allele at the *i*^th ^RV occurring in cases versus controls. *P_0 _*is equal to the proportion of cases among all subjects, where a similar proportion of the rare alleles at the RVs is expected to occur at random in the cases and the controls. A small *p *value indicates that the distribution of the rare alleles is not random.

A combination of protective, harmful, and neutral variants is likely associated with hypertension. Longitudinal hypertension data and whole genome sequence data were provided to the authors as part of the Genetic Analysis Workshop 18 (GAW18). The data set is from the Type 2 Diabetes Genetic Exploration by Next-generation sequencing in Ethnic Samples (T2D-GENES) Project 2, which was designed to identify RVs associated with hypertension and provided an opportunity to test a novel method for RV analysis.

The hypothesis of interest was to see whether significant RV associations occurred near CVs, and whether or not these associations were affected by the CVs occurring in genes versus not in genes. The RV analysis thus was related to and based off of single-marker association tests. Highly significant CVs were identified within chromosome 3, as were RVs around these locations. Based on a predetermined window size, these RVs were then analyzed with the C-alpha test to determine significant associations with hypertension. Markers in genes were compared to markers not in genes.

## Methods

Based on the hypothesis of interest, the analysis consisted of the following steps: data clean-up, single-marker association tests, and the RV analysis. The following sections provide details for each of these methods.

### Data clean-up

From the original GAW18 sequenced chromosome 3 file, only columns with the single-nucleotide polymorphism (SNP) IDs and genotype information for each subject were retained. Base-pair locations that appeared to have 3 or more alleles were excluded from further analyses. Only fully sequenced data from unrelated individuals was included in this analysis (*n *= 103). The real data rather than the simulated GAW18 data was used in order to not bias the results toward more significance in genes versus not in genes.

If a subject had hypertension listed for one or more of the four doctor visits, the subject was coded as 2 for affected. Otherwise, a subject was coded as 1 for unaffected. The covariates of interest were gender (M/F), age at first visit, smoking (Yes/No), and blood pressure medication (Yes/No). Similarly to how hypertension was coded, smoking was coded as 2 if the subject was listed as smoking for any of the 4 doctor visits, and subjects were coded as 2 if the subject was listed as using blood pressure medication during any of the 4 visits. Markers that did not satisfy the Hardy-Weinberg equilibrium (*p *<0.01) were excluded from the analysis.

### Single-marker association tests

Single-marker association tests were performed on CVs along chromosome 3. A total of 103 unrelated individuals were included in this analysis. The logistic models were adjusted for the following covariates: smoking, blood pressure medication, age at first visit, and gender. Because of large *p *values from the association tests, neither a significance threshold for the *p *value nor a correction for multiple testing was considered.

### Rare variant analysis

The top 10 significant CVs were chosen along chromosome 3 for further analysis. Five of these top 10 markers were located in genes. A SNP was considered to be within a gene if it was located anywhere within the 5′ and 3′ untranslated region (UTR) of the gene. Gene locations were based on information from GeneCruiser [7].

Window sizes of 1 kilobase (kb), 5 kb, and 25 kb were examined around these CVs for RVs. RVs were defined as having a MAF <0.05. These RVs were then extracted and a C-alpha test was calculated for each window size to determine the sensitivity of arbitrary window sizes. Singletons were removed from the analysis. The biased urn method was used to obtain C-alpha test *p *values that accounted for population stratification in permutations of case and/or control status [8]. From the GWAS simulated odd-numbered chromosomes, a reduced set of 39,883 SNPs with pairwise *r*^2 ^≤0.01 and no missing alleles was obtained. A total of 94 subjects were included in the reduced set of SNPs, of which 60 were cases and 34 were controls. The first 5 eigenvectors from a principal components analysis, along with the same covariates from above, were used to generate 1000 biased urn samples based on Fisher's noncentral hypergeometric distribution [8].

Data analyses were performed with PLINK version 1.07 and the R packages AssotesteR and Epstein et al's modified BiasedUrn package [8]. All data cleaning was performed with JMP version 10 and SAS version 9.2.

## Results

A total of 408,343 CVs were analyzed; 13,017 were nominally significant (*p *value for association test <0.05), and approximately 1% were located in genes. Table 1 contains the C-alpha test estimates and corresponding *p *values for both the association test and the C-alpha test for the top 5 markers in genes and the top 5 markers not in genes. The C-alpha test is sensitive to the specified window size, and for some markers an increase in window size corresponded to an increase in *p *values. For marker rs34366649, the opposite effect was seen, where the *p *value decreased as window size increased.

**Table 1:** C-Alpha test results for markers in genes versus markers not in genes

	Markers in genes					Markers not in genes				
		
Window	Marker	*p *Value*	# RVs tested	C-alpha statistic	Permuted *p *value^†^	Marker	*p *Value*	# RVs tested	C-alpha statistic	Permuted *p *value^†^
1 kb	35751033		5	−31.83	0.090			13	1.91	**0.008**
5 kb	(gene ID: 51185)	0.00064	27	−100.25	0.276	5020216	0.00141	50	−0.11	0.415
25 kb			154	−939.73	0.629			127	−35.27	0.935

1 kb	212007		6	1.29	0.207			1	n/a	n/a
5 kb	(gene ID: 2272)	0.00174	20	−2.14	0.437	3953247	0.00158	9	−33.10	0.091
25 kb			101	−27.57	0.485			53	−23.10	0.245

1 kb	262976		0	n/a	n/a			4	1.94	**<0.001**
5 kb	(gene ID: 55689)	0.00214	4	1.86	0.070	11719665	0.00173	23	16.35	**0.037**
25 kb			34	10.96	**0.002**			64	23.23	**0.041**

1 kb	53856668		7	−0.42	0.367			4	−2.22	0.407
5 kb	(gene ID: 2272)	0.00192	22	−2.80	0.507	4553960	0.00186	10	−4.37	0.440
25 kb			104	−62.61	0.329			47	−1.37	**0.009**

1 kb	1464118		1	n/a	n/a			2	−6.40	0.227
5 kb	(gene ID: 8626)	0.00220	3	−1.28	0.891	34366649	0.00222	9	−5.46	0.161
25 kb			27	−25.29	0.658			15	36.97	**0.030**

n/a, Not available.

* From single marker association test.

^†^Based on 1000 permutations using biased urn method. Highlighted p values are significant at an alpha of 0.05.

On average, markers not in genes were more significant than markers in genes. Fisher's exact tests comparing significance for the 1-kb, 5-kb and 25-kb windows were not significant (*p *= 0.4667, *p*=1 and *p *= 0.5238, respectively).

## Conclusions

Our hypothesis examined whether or not important RVs are near significantly associated CVs based on genetic distance. Our RV analysis thus was related to and based off of single-marker association tests. Highly significant CVs were identified within chromosome 3, as were RVs around these locations. Based on a predetermined window size, these RVs were then analyzed with the C-alpha test to determine significant associations with hypertension.

Based on our results, we found significant RVs around highly significant CVs. However, we also found that the C-alpha test was sensitive to the specified window size. Future research can examine more closely how the specified window size affects the C-alpha test.

When comparing markers in genes to markers not in genes, we found that markers not in genes had more significant C-alpha test *p *values than markers in genes. These findings were not significant with a Fisher's exact test. Very few CVs that were analyzed occurred in genes (approximately 1%), and this may have biased the results. In addition, the *p *values from the single-marker association tests were underpowered (the smallest *p *value was 0.00064). However, the *p *values from the single-marker association tests were comparable in magnitude in genes versus not in genes. Also, among the top 10 most significant SNPs, 5 were located in genes and 5 were located not in genes, which minimized bias in the results. Future research can investigate in more depth whether RVs occur more in genes versus not in genes.

## Competing interests

The authors declare that they have no competing interests.

## Authors' contributions

AF, AW, and LS designed the overall study. AF conducted statistical analyses and drafted the manuscript. AP and LS conducted data cleaning. All authors read and approved the final manuscript.
